# Foot and mouth disease vaccine efficacy in Africa: a systematic review and meta-analysis

**DOI:** 10.3389/fvets.2024.1360256

**Published:** 2024-06-06

**Authors:** Ashenafi Kiros Wubshet, Gebremeskel Mamu Werid, Teshale Teklue, Luoyi Zhou, Chimedtseren Bayasgalan, Ariunaa Tserendorj, Jinjin Liu, Livio Heath, Yuefeng Sun, Yaozhong Ding, Wenxiu Wang, Alexei D. Zaberezhny, Yongsheng Liu, Jie Zhang

**Affiliations:** ^1^State Key Laboratory of Veterinary Etiological Biology, National/OIE Foot and Mouth Disease Reference Laboratory, Lanzhou Veterinary Research Institute, Chinese Academy of Agricultural Sciences, Lanzhou, China; ^2^Department of Veterinary Basics and Diagnostic Sciences, College of Veterinary Science, Mekelle University, Mekelle, Tigray, Ethiopia; ^3^Davies Livestock Research Centre, School of Animal & Veterinary Sciences, University of Adelaide, Roseworthy, SA, Australia; ^4^Mekelle Agricultural Research Center, Mekelle, Ethiopia; ^5^Hebei Key Laboratory of Preventive Veterinary Medicine, College of Animal Science and Technology, Hebei Normal University of Science and Technology, Qinhuangdao, China; ^6^School of Veterinary Medicine, Mongolian University of Life Sciences, Ulaanbaatar, Mongolia; ^7^Transboundary Animal Diseases Programme, Onderstepoort Veterinary Research, Agricultural Research Council, Onderstepoort, South Africa; ^8^Shandong Binzhou Animal Science and Veterinary Medicine Academy, Binzhou, China; ^9^Federal State Budgetary Scientific Institution “All-Russian Research and Technological Institute of Biological Industry” (FSBSI VNITIBP), Moscow, Russia

**Keywords:** FMDV, FMD vaccine, efficacy, effectiveness, Africa

## Abstract

**Background:**

Several factors, such as diverse serotypes, vaccination methods, weak biosecurity, and animal movements, contribute to recurrent Foot-and-Mouth Disease Virus (FMDV) outbreaks in Africa, establishing endemicity. These outbreaks cost over $2 billion annually, prompting a high-priority focus on FMDV vaccination. Despite extensive efforts, vaccine efficacy varies. This study aims to evaluate routine foot and mouth disease (FMD) vaccines in Africa via systematic review and meta-analysis.

**Methods:**

A systematic review and meta-analysis were carried out following Preferred Reporting Items for Systematic Reviews and Meta-Analyses (PRISMA) guidelines. Meta-analysis was conducted to assess the efficacy of FMDV vaccination using the meta for package of R.

**Results:**

Vaccinated animals have roughly a 69.3% lower chance of FMDV infection compared to unvaccinated animals, as indicated by the pooled results from the random-effects model, which showed a risk ratio (RR) of 0.3073. There was a statistically significant heterogeneity (*p* < 0.05) across all of the included articles.

**Conclusion:**

Overall findings suggest that if properly planned and implemented, FMDV vaccination programs and strategies in Africa could help control the spread of the disease throughout the continent and beyond.

## 1 Introduction

Foot and mouth disease (FMD) represents a significant economic challenge, particularly in regions where it is endemic. Globally, the disease causes economic losses estimated between US$6.5 to $21 billion annually in endemic areas, with FMD-free countries and zones also incurring costs exceeding US$1.5 billion per year (1). In Africa, the impact of the disease is particularly severe, causing annual economic losses of more than $2 billion (2). The global market for Foot and Mouth Disease Virus (FMDV) vaccines was valued at $1.4 billion in 2022 and is projected to rise to over US$2.4 billion by 2031 (3). Eradicating FMD from a country is often considered the ultimate goal in managing this disease, leading to a longstanding reliance on inactivated viral vaccines as a primary control measure (4).

However, the efficacy of these vaccines is challenged by the significant antigenic variability of FMDV serotypes and the typically short-lived immunity they provide (5). The Organization for World Animal Health (WOAH) has categorized the seven known FMDV serotypes into three distinct pools for Africa, aiming to enhance the management of FMD control programs in a global context (6–8). The evolving nature of these viruses in Africa requires ongoing adaptation in vaccine development, potentially affecting vaccine efficacy. Polyvalent vaccines, targeting multiple serotypes, are commonly used in Africa, which may inadvertently contribute to the evolution of the virus. The endemic status of FMDV in Africa is exacerbated by factors such as multiple serotypes, inadequate biosecurity measures, contact of livestock with wildlife (9), and transboundary animal movements, necessitating a comprehensive control strategy (5).

Vaccination is crucial in controlling FMD globally. However, FMD control in Africa is complex due to the unique epidemiology of the disease in the region. The presence of six of the seven FMD serotypes, with wide antigenic variations, requires constant vaccine virus changes (5, 10). Moreover, the use of vaccination and movement control as control measures is hindered by the disease’s persistence and the economic implications of FMD outbreaks (11, 12). Despite lacking a one-size-fits-all approach for FMD control in Africa, vaccination remains a primary strategy for governments and livestock producers across the continent. This growing demand for FMDV vaccination is challenged by the limited capacity of the continent to produce sufficient vaccines, indicating a nascent stage in the animal vaccine market in Africa (3). Practices involving routine and emergency vaccinations are common, with quality control overseen by entities like the African Union Pan African Veterinary Vaccine Centre (AU-PANVAC) in Ethiopia (13). The efficacy of FMDV vaccines is contingent on understanding the prevalent serotypes, matching field and vaccine strains, and ensuring the potency of the vaccine in inducing a durable immune response (14). Additionally, the success of vaccination efforts is influenced by the specifics of regional vaccination programs, the implementation of routine vaccine matching tests, the types of adjuvants used, and the scale of animal immunization during campaigns (5).

Considering these complexities, understanding the factors affecting vaccine efficacy, the state of vaccination efforts, and the effectiveness of current vaccines is crucial for the concerted effort to control, prevent, or eliminate FMDV in Africa. Therefore, this systematic review and meta-analysis were conducted to analyze existing literature on the efficacy of routine FMD vaccines in Africa, filling a gap in systematic evaluations of these vaccines.

## 2 Materials and methods

### 2.1 Literature search and selection

The search strategy, designed in accordance with the Preferred Reporting Items for Systematic Reviews and Meta-Analyses (PRISMA) guidelines, aimed to capture studies pertinent to the efficacy of FMD vaccines in Africa (15). The population targeted included livestock such as cattle, sheep, goats, and pigs within African countries. The intervention of interest was defined as the administration of FMD vaccine to livestock species in Africa. It included all types FMD vaccination programs practiced in Africa, covering a range of vaccine types, regimens, and delivery methods. Comparative analysis was carried out between vaccinated and unvaccinated populations and among various vaccination strategies, whenever data allowed. The risk ratio (R.R.) of FMD incidence was used as a primary outcome measure. Both experimental and observational study designs—randomized controlled trials, case–control and cohort studies were included in the study.

To identify studies relevant to FMD in domestic livestock species within African regions, focusing on antibody titer post-vaccination and utilizing standard immunological assays, a comprehensive literature search was conducted across PubMed, Web of Science, and Scopus databases. The search covered all articles published until 19/05/2023. The search strings for each database were: PubMed: (“foot and mouth disease”[Title/Abstract] OR FMD*[Title/Abstract]) AND (cattle[Title/Abstract] OR bovine[Title/Abstract] OR “*Bos taurus*”[Title/Abstract] OR goat[Title/Abstract] OR caprine[Title/Abstract] OR “*Capra aegagrus* hircus”[Title/Abstract] OR sheep[Title/Abstract] OR ovine[Title/Abstract] OR “*Ovis aries*”[Title/Abstract] OR pig[Title/Abstract] OR porcine[Title/Abstract] OR swine[Title/Abstract] OR “*Sus scrofa domesticus*”[Title/Abstract]). In Web of Science: TS = (“foot and mouth disease” OR FMD*) AND (TS = (cattle) OR TS = (bovine) OR TS = (“*Bos taurus*”) OR TS = (goat) OR TS = (caprine) OR TS = (“*Capra aegagrus* hircus”) OR TS = (sheep) OR TS = (ovine) OR TS = (“*Ovis aries*”) OR TS = (pig) OR TS = (porcine) OR TS = (swine) OR TS = (“*Sus scrofa domesticus*”)). In Scopus: TITLE-ABS-KEY(“foot and mouth disease” OR FMD*) AND (TITLE-ABS-KEY(cattle) OR TITLE-ABS-KEY(bovine) OR TITLE-ABS-KEY(“*Bos taurus*”) OR TITLE-ABS-KEY(goat) OR TITLE-ABS-KEY(caprine) OR TITLE-ABS-KEY(“*Capra aegagrus* hircus”) OR TITLE-ABS-KEY(sheep) OR TITLE-ABS-KEY(ovine) OR TITLE-ABS-KEY(“*Ovis aries*”) OR TITLE-ABS-KEY(pig) OR TITLE-ABS-KEY(porcine) OR TITLE-ABS-KEY(swine) OR TITLE-ABS-KEY(“*Sus scrofa domesticus*”)).

The Covidence platform^1^ was used to facilitate the screening and extraction process of the collected studies. Primarily, the title and abstract and then the full text of each article were screened for relevance based on the inclusion and exclusion criteria (Table 1). Each of the collected articles was screened by three co-authors (AKW, GMW, and TTA), and conflicts in screening were resolved either by a third senior author or through consensus among the three authors.

**Table 1:** Inclusion and exclusion criteria used for screening articles.

Criteria	Inclusion criteria	Exclusion criteria
Location	Studies conducted exclusively within African regions.	Studies originating from regions outside Africa.
Study Type	Case control, cohort or randomized controlled trials.	Studies of other types include cross-sectional, case reports, reviews, or opinion pieces.
Species	Livestock species (cattle, sheep, goat, and swine) were clearly identified in the study.	Studies with unspecified or mixed species without clear identification.
Farm Type	Studies providing detailed information on farm or production conditions.	Studies with insufficient or vague descriptions of farm types and conditions.
Testing Method	Use of World Organization for Animal Health (WOAH) approved or regionally standard immunological assays.	S Studies employing non-standardized or unapproved testing methods.
Objectives	Objectives focused on evaluating antibody titer to FMD post-vaccination or following natural infection.	Studies primarily centered on comparative field trials for vaccine efficacy. Investigations into antibody response dynamics without a specific focus on post-vaccination efficacy.
Outcomes	Reporting the number of animals with sufficient immunity and the total samples tested in both vaccinated and unvaccinated groups	Studies that lack clear criteria for determining sufficient immunity based on standard guidelines. Studies where the vaccine type remains unidentified.

### 2.2 Inclusion and exclusion criteria

The studies were eligible for inclusion in our systematic review if they fulfilled the criteria listed in Table 1. This systematic and meta-analysis study excluded studies that did not take place in Africa, studies that did not use an FMDV vaccine as an intervention, studies that used animal populations of unspecified or mixed species, review articles, meta-analyses, news reports, and any research lacking data that can be extracted. The details of the exclusion criteria are listed in Table 1. The included studies were further categorized for qualitative and quantitative analyses based on the study design. For quantitative analysis, studies that provided detailed data on the number of animals vaccinated, the number of animals unvaccinated, and the immune response observed in each group were selected. Studies included in the final screening that lacked specific data on the number of animals vaccinated, the number of animals unvaccinated, and the observed immune responses in each group were allocated only to the qualitative analysis.

### 2.3 Data extraction and quality assessment

Data were extracted using Covidence, including author information, publication year, animal species, country, farming system, study design, sample type, species, vaccine type, and FMD detection method. The total number of animals or herds tested, FMD-positive cases, and age distribution were also collected. For articles included in qualitative analysis, the Joanna Briggs Institute Qualitative Assessment and Review Instrument (JBI-QARI)^2^ with slight modification was used to assess article quality.

In the systematic review and meta-analysis of FMD vaccine trials in Africa, the Risk of Bias in Non-randomized Studies of Interventions (ROBINS-I) tool was employed to evaluate the risk of bias in non-randomized studies (16). This tool examined seven domains: risk of bias due to confounding, selection of participants, classification of interventions, deviations from intended interventions, missing data, measurement of outcomes, and selection of the reported results. Two independent reviewers assessed these domains, categorizing them as having low, moderate, serious, or critical risk of bias or no information. A third independent reviewer resolved discrepancies. A study was considered to have an overall low risk of bias if all domains were judged as low risk and as having a critical risk of bias if any domain was judged as high risk. The Cochrane Risk of Bias 2.0 tool was intended for randomized trials (17).

### 2.4 Statistical and meta-analysis

The meta-analysis was conducted using the random effects model (18), which is implemented in the metabin function in R (19). The primary measure of interest was RR, which compares the risk of an event which is the occurrence of FMD among the study subjects, (vaccinated and unvaccinated groups). The pooled risk ratio was represented with a 95% confidence interval. The ability of the vaccine to provide protection against FMD in vaccinated populations compared to unvaccinated ones, referred to here as FMD vaccine efficacy (20), was measured using vaccine efficacy, VE, which is calculated as 1-RR (21). RR was calculated as:


$$RR=\frac{\mathrm{Risk}\;\mathrm{in}\;\mathrm{the}\;\mathrm{vaccinated}\;\mathrm{group}}{\mathrm{Risk}\;\mathrm{in}\;\mathrm{the}\;\mathrm{unvaccinated}\;\mathrm{group}}$$

Where:


$$\begin{array}{l} \mathrm{Risk}\;\mathrm{in}\;\mathrm{the}\;\mathrm{vaccinated}\;\mathrm{group}=\\ \frac{\mathrm{Number}\;\mathrm{of}\;\mathrm{vaccinated}\;\mathrm{animals}\;\mathrm{that}\;\mathrm{were}\;\mathrm{not}\;\mathrm{protected}}{\mathrm{Total}\;\mathrm{number}\;\mathrm{of}\;\mathrm{vaccinated}\;\mathrm{animals}} \end{array}$$

$$\begin{array}{l} \mathrm{Risk}\;\mathrm{in}\;\mathrm{the}\;\mathrm{unvaccinated}\;\mathrm{group}=\\ \frac{\mathrm{Number}\;\mathrm{of}\;\mathrm{unvaccinated}\;\mathrm{animals}\;\mathrm{that}\;\mathrm{tested positivive for}\;FMD}{\mathrm{Total}\;\mathrm{number}\;\mathrm{of}\;\mathrm{unvaccinated}\;\mathrm{animals}} \end{array}$$

NVivo 20.0 software (22) was used for systematic identification, coding, and thematic categorization of risk factors and variables. Each of the included studies for qualitative analysis was analyzed based on thematic areas such as vaccine type, adjuvant type, risk factor type, vaccination strategy and country.

## 3 Results

### 3.1 Study characteristics

**Figure 1:**
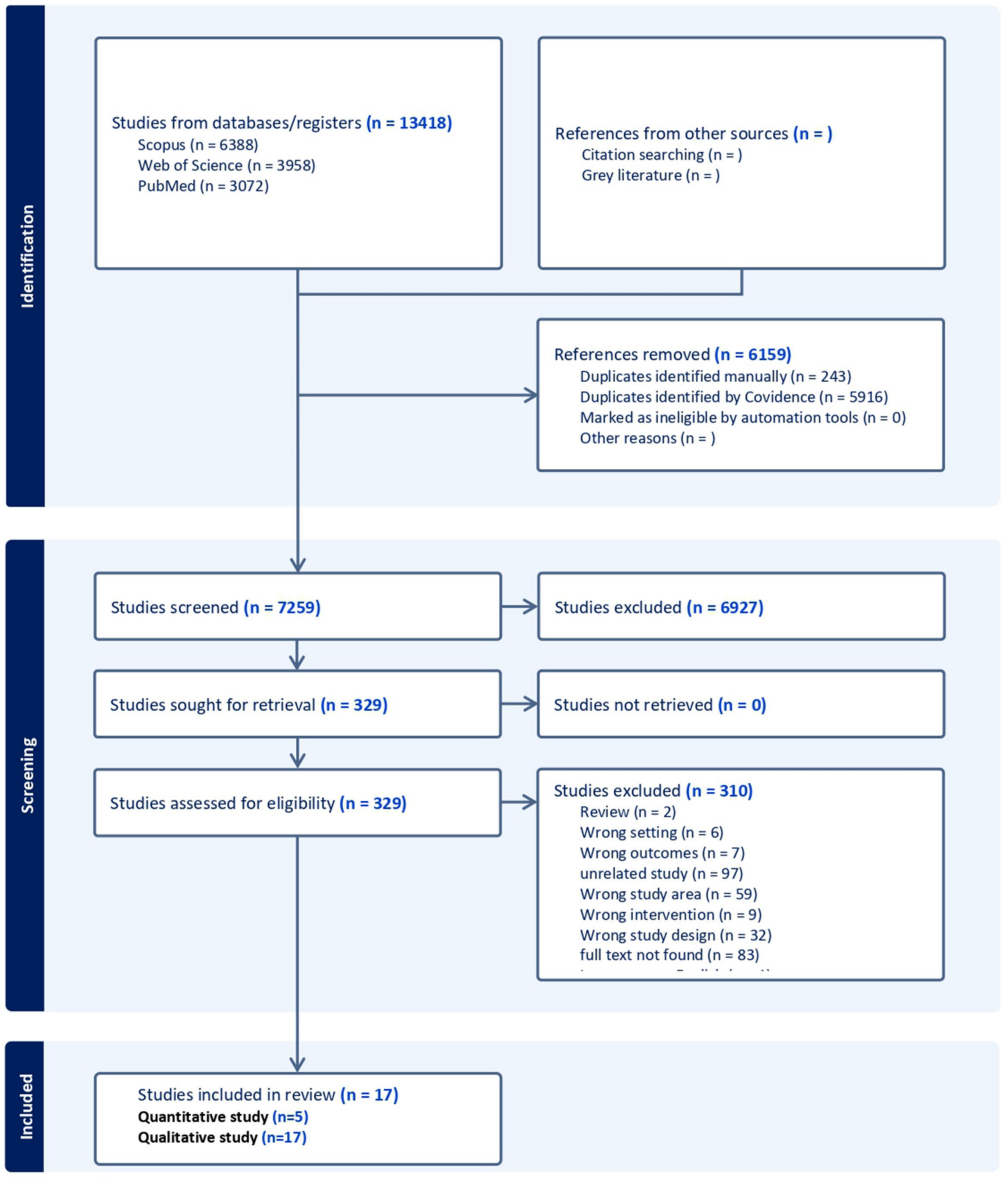
Steps followed for data collection and screening.

Initially, a total of 13,418 articles were collected. After removing duplicates, we screened 7,259 articles at the title and abstract screening stage. At the full-text level, 329 studies were assessed for eligibility. During the full-text-based screening, 21 studies were included for data extraction. After data extraction and quality assessment, three studies were excluded due to data quality or methodology, leaving only 17 studies for final data analysis. Although all included studies were assessed for bias, none met the criteria for exclusion based on the results of this bias assessment. Among these, 17 were included in the qualitative analysis, while 14 articles initially met the eligibility criteria for the quantitative analysis; nine of these articles were excluded due to having either a zero RR or a zero in the denominator during the RR calculation. As a result, only five studies were ultimately included in the meta-analysis (Figure 1).

Information about each study, including the year of publication, country of study, study design, duration of study, species of animals, breeds of animals, and age group, is displayed in Supplementary Table 1. Details regarding the FMDV vaccine type, the number of vaccinated and unvaccinated individuals, the percentage of effective interventions that resulted in seroprotection, the vaccine serotype, the serotypes circulating in the field, and the specific immune assay used were also recorded (Supplementary Table 2).

The dataset comprising 17 articles offered an analysis of FMD vaccine efficacy in Africa, covering a period from 1996 to 2023. The majority of these studies occurred in South Africa and Egypt, and they used a variety of research methodologies, such as randomized controlled trials, cohort studies, and case–control studies, and lasted from 21 days to 40 weeks. Most of these studies were on bovine species, particularly local breeds. However, some studies also included ovine and porcine species. Most of the bovine species included in the study came from dairy farms, where they were raised from birth, while the others were likely introduced from elsewhere.

The studies predominantly used inactivated vaccines, incorporating specific serotypes like O, A, and SAT2 and explored a broad spectrum of vaccination aspects, such as efficacy, potency, immune response, and the influence of adjuvants. The size of study populations varied, ranging from small groups of around 4 animals to larger cohorts involving up to 191 animals. Moreover, the protection rates post-vaccination showed variation, with some studies reporting complete protection (100%) in certain cases.

These studies also provided insights into the prevalence of FMD in unvaccinated animals, demonstrating the incidence of positive test results. Methodologically, a range of techniques, including serum neutralization test (SNT), ELISA, and RT-qPCR, were employed to detect and analyze the virus and assess the immune response. Overall, the objectives of these studies were multifaceted, primarily focused on understanding the efficacy of routine vaccinations, the immune response in vaccinated animals, and assessing the performance of different vaccine formulations in diverse field conditions.

### 3.2 Diversity of FMDV serotypes and vaccine variability in Africa

It was found that in Africa, the most widely used form of FMD vaccine was an inactivated viral vaccine combined with an adjuvant, except in one study (23), a reverse genetically synthesized vaccine. In all these studies, the vaccines were designed to provide immunity against the following specific FMD virus serotypes: O, A, SAT 1, SAT 2, and SAT 3. Cameroon is home to four different FMD virus serotypes (FMDV; O, A, SAT1, SAT2) thus, a trivalent inactivated vaccination carried out to combat the three most common serotypes—O, A, and SAT2 (24). Similarly, Egypt and Kenya have also been experiencing these serotypes (25). According six studies, one from Cameron (24) and five from Egypt (26–30), the O, A, and SAT2 FMDV serotypes were found prevalent in the field which had correspondence to the vaccine strain. In these studies, the trivalent inactivated vaccine consisting of O, A, and SAT2 was used during efficacy testing. On the other hand, vaccine efficacy trial against A and O FMDV serotype was employed in three studies (31, 32) in Egypt and (33) in South Africa despites the field strains are (O, A, and SAT2) and SAT1, SAT2 and SAT3 FMDV serotypes, respectively, in these regions. In some studies in Egypt (26, 34, 35) vaccine efficacy evaluation was conducted against O serotype. In South Africa, only SAT serotypes were widespread, and vaccine tests showed trivalent inactivated vaccine against SAT1, SAT2, and SAT3 of distinct strains (M. CLOETE, 2008) (36), Furthermore, the efficacy test for one vaccine also targeted a monovalent vaccine specifically designed to combat the SAT2 serotype (23). However, vaccination strategies and programs vary by country; these key highlights are presented below.

#### 3.2.1 FMDV dynamics and vaccination strategies in Egypt

FMV is endemic in Egypt and Three FMDV serotypes: O, A, and SAT2 have been reported (29). Three different vaccines are used against FMDV in Egypt. Among these are locally manufactured vaccines, including inactivated serotypes A, O, and SAT2 of FMDV. A multivalent commercial vaccine (Merial, France) also covers six serotypes. The national FMDV control program predominantly employs the two locally produced vaccines, which are administered biannually. Despite this, vaccination coverage remains suboptimal (37).

The introduction of the FMD control strategy has led to a significant reduction in FMD cases. However, outbreaks are still being reported, even in areas where immunization is done regularly (30). In the Kafrelsheikh Governorate of Egypt, both vaccinated and non-vaccinated cattle have become sick or died from FMDV (37). A study of the efficacy of a commercial local trivalent FMD vaccine against recently isolated O-EA3 showed that a protective neutralizing serum antibody titer of 1.2 log10 started to develop from the third-week post-vaccination, reaching a 156 log10 titer against FMDV type O in vaccinated calves. The challenge test, conducted on the 28th day post-vaccination using the O-EA3 virus, revealed a 100% protection level in vaccinated calves (38).

The available local commercial inactivated FMDV vaccine batches, isolate serotypes O/EGY/4/2012, A/EGY/1/2012, and SAT2/EGY/2/2012, are impotent and not effective against the current circulating FMDV field isolate SAT2 topotype VII, Lib-12 lineage (29). Another study carried out on cattle from the Sharkia Governorate of Egypt showed that FMDV isolates from vaccinated and non-vaccinated groups were similar but did not match the local vaccine strain (39). A study that primarily focused on testing various immunization strategies against FMDV in cattle using two vaccine formulations, the Montanide ISA 206 oil-based inactivated vaccine and the Montanide IMS 1313 semi-purified mucosal vaccine, found differing levels of efficacy. Intranasal administration of the mucosal vaccine led to IgA production in nasal and salivary secretions and offered 20 to 40% protection, depending on whether one or two doses were given. In contrast, the inactivated vaccine alone achieved 80% protection. However, a prime-boost strategy combining initial mucosal vaccination followed by the inactivated vaccine proved most effective, delivering 100% protection against FMDV in cattle (40).

#### 3.2.2 FMDV dynamics and vaccination strategies in Kenya

In a survey conducted in Kenya, serological evidence for SAT 1 FMDV infection in vaccinated and non-vaccinated pigs was found without obvious clinical signs during FMD outbreaks in cattle. Specifically, among the 191 collected serum samples, 42 originated from pigs immunized against FMDV serotypes O, A, and SAT2. However, a significant majority (92 out of 101) of the samples that tested positive exhibited antibodies identifiable through serotype-specific ELISAs, primarily targeting the SAT1 serotype, with only five instances of high antibody titers against SAT1 in the vaccinated group (41).

#### 3.2.3 FMDV dynamics and vaccination strategies in Cameron

In Cameroon, all vaccinated animals remained clinically healthy during the study, whereas clinical signs of FMD were reported in six non-vaccinated animals (24). Moreover, neither the frequency of FMDV RNA in oropharyngeal fluid (OPF) nor the seroconversion rate were correlated with vaccination status. However, the likelihood of detecting FMDV RNA in OPF was higher in younger cattle than in older animals (24).

#### 3.2.4 FMDV dynamics and vaccination strategies in South Africa

In South Africa, a vaccination program using an inactivated trivalent FMD vaccine (SAT1, SAT2 and SAT3) targeting the wildlife-livestock interface along the western border of Kruger National Park was found to have adequate seroconversion in a high proportion of vaccinated cattle with a relatively short-lived humoral response (42). In a different study, cattle were administered a multivalent vaccine composed of SAT 1A, SAT 1B, SAT 2A, SAT 2B, and SAT3 serotypes, resulting in seroconversion beginning as early as 7 days post-vaccination, exhibiting a range of immune responses (23). The study also found that clinical protection lasted up to 12 months when either one or two booster vaccinations were given within the first 6 months following the initial vaccination. The difference in efficacy between administering one or two booster shots was minimal (23). A study that evaluated the immune responses of stabilized SAT2 antigens of FMDV in cattle showed that administering two doses of the SAT2 antigen, augmented with ISA206B adjuvant, at intervals of 4–6 weeks, conferred effective protection to cattle for a duration of up to 5 months post-vaccination. After the initial vaccination, there was a marked difference in both total and neutralizing antibodies, especially in the vSAT2-93H group, compared to other groups that received the vaccine. However, this disparity in antibody response diminished and was no longer significant after administering the second vaccine dose (33).

### 3.3 Efficacy of adjuvants in enhancing FMDV vaccine performance

Using oil-based adjuvants or adjuvants with oil in FMD vaccines may be more effective than the conventional aluminum hydroxide gel and saponin adjuvants, as they result in higher immune responses in cattle (43). One of the six cattle vaccinated with the ISA 50 vaccine was protected from live virus challenge despite the presence of neutralizing antibodies to the SAT 2B antigen at the time of challenge. SA 206B-adjuvanted FMD vaccine without saponin protected pigs against live heterologous virus challenge 36 days post-vaccination (44). In Egypt, the evaluation of three different types of vaccines in sheep revealed that the double oil emulsion–FMD vaccine using Spane 80 as emulsifier showed much higher antibody titers and longer duration of immunity than the other two vaccine preparations (34). In South Africa, the mean titers of three oil-based preparations were higher than those induced by the Alhydrogel and Saposin-based vaccines (36). A single administration of the heptavalent ISA 206 VG oil-adjuvanted vaccine prepared from A-Africa-IV, A-Iran05, O-Manisa, O-PanAsia2, SAT-2 LIB-12 and O-EA3, SAT-2 Gharbia, strains resulted in a high mean neutralizing antibody titer 28 days post-vaccination (30).

### 3.4 Challenges and efficacy of FMD vaccination programs in Africa

African FMDV vaccination programs have faced various challenges and exhibited mixed results across different regions. In Kenya, there have been instances of FMD outbreaks in both vaccinated and unvaccinated cattle (41). In Egypt, local commercial vaccines showed protective effects, but there were observations of disease occurrence in both vaccinated and non-vaccinated animals (29, 39). Since export has never been an objective of regional livestock raising in Cameroon, the country has never had a structured FMD control program (24). In South Africa, FMD vaccination has been a critical component of disease control, especially given the country’s diverse wildlife reservoirs that can harbor the virus. South Africa uses a zoning strategy, with specific areas designated as FMD-free zones where vaccination is not routinely practiced. In contrast, vaccination is more common in other areas, particularly those bordering wildlife reserves or neighboring countries with endemic FMD. The effectiveness of vaccination programs in South Africa and other African nations often hinges on several factors: the selection of appropriate vaccine strains to match circulating serotypes, efficient vaccine distribution and administration, and regular surveillance and monitoring. However, challenges such as logistical hurdles, limited resources, and the presence of multiple FMDV serotypes complicate these efforts.

### 3.5 Meta-analysis of the FMDV vaccine efficacy

In this meta-analysis, the findings from five studies were pooled to assess the efficacy of FMDV vaccination. The studies included in our analysis were (23, 25, 37, 41, 44). The pooled results from the random-effects model suggested RR of 0.31 and a VE of 0.69 (69%). The analysis showed that the FMD vaccination reduced the risk of FMDV infection by approximately 69% in the vaccinated group compared to the unvaccinated group.

**Figure 2:**
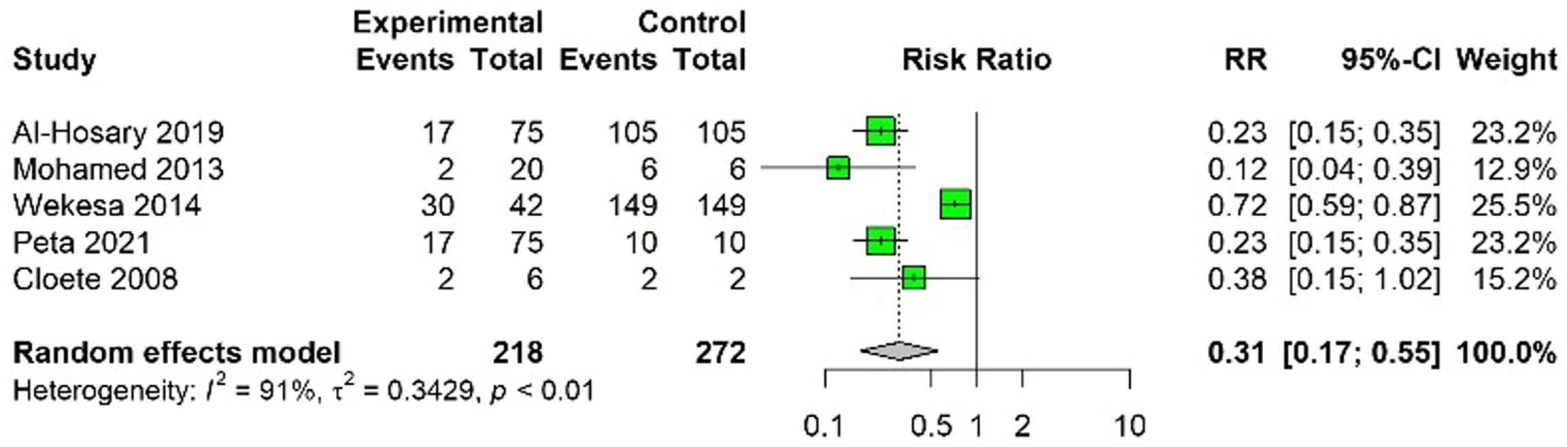
Forest of Foot-and-Mouth Disease (FMD) vaccination efficacy studies expressed as RR of FMD in vaccinated versus unvaccinated populations. A green square represents each study, the size of which correlates with the study’s weight in the meta-analysis. The horizontal lines crossing each square depict the 95% confidence intervals (CIs) for the RR of each study.

A higher level of heterogeneity was observed among the studies analyzed in the present findings (Figure 2). Test for heterogeneity yielded a Q-value of 46.97 (with 4 degrees of freedom), which was statistically significant (*p* < 0.05), confirming the presence of significant variability among the included studies.

## 4 Discussion

The current study explored the efficacy of FMD vaccinations in Africa. The pooled results from the meta-analysis indicated that vaccinated animals have a substantially lower chance of FMDV infection compared to unvaccinated ones, with a VE of 0.69 (69.3%). This finding underscores the potential of vaccination programs to reduce the prevalence of FMDV in livestock.

However, the observed high level of heterogeneity among the studies included in the meta-analysis highlights the variability in vaccine efficacy across different settings and conditions. This variability could be attributed to several factors, including the diversity of FMDV serotypes in Africa, the varying quality of vaccines used, and differences in vaccination regimes (45). The match between vaccine strains and circulating field strains, in particular, emerges as a crucial determinant of vaccine efficacy (46). FMD is widespread across many African countries, with varying prevalence influenced by factors such as geographical location, livestock management practices, and the effectiveness of control measures (47–49). As it has shown in these studies, multiple vaccines are employed in response to the diverse serotypes of FMD in Africa. These vaccines are formulated to target the most prevalent serotypes in specific regions (50). Meanwhile, the circulating strains in each country determine the vaccine choice and its serotype coverage (51).

The current study showed that vaccines for serotypes: O, A, SAT 1, SAT 2, and SAT 3 have been used in Africa. However, the diversity of FMDV serotypes in Africa, particularly the variability within serotypes O, A, SAT1, SAT2, and SAT3, presents a substantial challenge to vaccination efforts (52). This diversity is characterized by the presence of multiple serotypes in the continent and the significant genetic heterogeneity within each serotype, which has critical implications for the development and deployment of vaccines. For instance, the co-circulation of highly divergent lineages within the SAT2 serotype in regions like Kenya demonstrates the dynamic nature of FMDV evolution (53). This evolution is not confined to a single serotype; the ongoing phylodynamics of serotypes A and SAT2, including recent isolates from Cameroon, highlight the rapidly changing landscape of FMDV in Africa (54). Such genetic diversity within serotypes requires constant monitoring and characterization of field strains to ensure that the vaccines used are effective against the currently circulating strains. Meanwhile, the circulating strains in each country determine the vaccine choice and its serotype coverage (51). Moreover, significant genetic heterogeneity was found in the leader and P1-coding regions of serotypes A and O (55, 56). This heterogeneity suggests the potential for genetic recombination, which could lead to the emergence of new strains with different antigenic properties, further complicating vaccine design and efficacy.

Interestingly, the current study demonstrated the role of adjuvants in enhancing vaccine performance. Oil-based adjuvants have been shown to enhance immune responses and protective efficacy in cattle when used in FMD vaccines (57–62). These adjuvants, such as Montanide ISA-201 and ISA 61 VG, have been found to elicit higher immune responses and provide better protection levels compared to conventional adjuvants. Therefore, the use of these adjuvants in FMD vaccine formulations could be a potential avenue for improving vaccine performance.

Vaccination programs play an important role in controlling or preventing FMD globally. However, in Africa, vaccination programs vary in design and implementation. Prophylactic vaccination can greatly reduce the potential for major FMD epidemics, while reactive vaccination and culling strategies can help control ongoing outbreaks (63). Moreover, the effectiveness of vaccination can be influenced by factors such as contact rates between wildlife and livestock (9), and the presence of different FMD virus strains (5) and fast replication rate, transmissibility, and antigenic diversity of the FMD virus (64). Inadequate epidemiological understanding and control measures also pose challenges to FMD control (12). The introduction of FMD into FMD-free regions, such as North Africa and Europe, underscores the need for strong intervention strategies (65). Key challenges include the diversity of the FMD virus, inadequate monitoring systems, varied animal species and farming practices, and technical issues like maintaining the cold chain (66). Even with effective vaccines, outbreaks in vaccinated populations suggest problems such as insufficient coverage, strain mismatches, and waning immunity (52). As highlighted in studies by Katie Lloyd-Jones, Woldemariyam (52, 66), and Ayelet (67), these issues, along with uncontrolled animal movement and weak biosecurity, complicate FMD control. Hence, regular updates and adaptations to vaccination strategies are vital, considering the dynamic nature of FMDV serotypes.

Moreover, the effectiveness of vaccination programs hinges on several operational factors, including efficient vaccine distribution, regular surveillance and monitoring, and the selection of appropriate vaccine strains (68). The challenges African countries face in maintaining a consistent supply of effective vaccines, even for humans (69), often due to financial constraints and logistical issues, further complicate these efforts.

In light of these findings, future research should focus on developing multivalent vaccines capable of providing broad protection against multiple serotypes prevalent in Africa. This may involve exploring safer and more effective second and third-generation vaccines against a wide variety of FMDV serotypes. Additionally, studies exploring the longevity of immunity conferred by different vaccine formulations and the efficacy of different vaccination regimes would be invaluable. Understanding the dynamics of FMDV serotypes and their evolution in response to vaccination pressures is also crucial for effectively managing FMD in Africa.

## 5 Conclusion and future perspective

While routine FMD vaccination programs in Africa show promise in controlling the disease, their effectiveness is influenced by multiple factors ranging from vaccine quality and serotype specificity to operational and logistical challenges. A multifaceted approach involving improved vaccine formulations, introducing improved vaccine designing approaches, tailored vaccination strategies, enhanced surveillance systems and continuous and harmonized vaccine efficacy studies is essential for effectively controlling and eventually eradicating FMD from Africa.

## Data availability statement

The original contributions presented in the study are included in the article/Supplementary material, further inquiries can be directed to the corresponding authors.

## Author contributions

AW: Conceptualization, Data curation, Methodology, Writing – original draft, Writing – review & editing, Formal analysis. GW: Formal analysis, Methodology, Software, Writing – original draft, Writing – review & editing. TT: Data curation, Methodology, Writing – review & editing, Formal analysis. LZ: Writing – review & editing. CB: Writing – review & editing, Visualization. AT: Writing – review & editing. JL: Writing – review & editing. LH: Resources, Validation, Writing – review & editing. YS: Resources, Validation, Writing – review & editing. YD: Resources, Validation, Writing – review & editing. WW: Resources, Validation, Writing – review & editing. AZ: Resources, Writing – review & editing. YL: Project administration, Supervision, Validation, Writing – review & editing. JZ: Project administration, Supervision, Validation, Writing – review & editing.

## Footnotes

^1^https://app.covidence.org/

^2^https://jbi.global/critical-appraisal-tools
