# Supplementary material for: Foot and mouth disease vaccine efficacy in Africa: a systematic review and meta-analysis
Source: Front Vet Sci. 2024 Jun 6;11:1360256. doi: 10.3389/fvets.2024.1360256 (PMC11187330; doi:10.3389/fvets.2024.1360256)
Supplement: Supplementary file 1 [file Table_1.pdf]

Supp Table 1. Presents the qualitatively data extracted from 17 published papers that satisfy the selection criteria, which focus on the vaccine efficacy against Foot-and-Mouth Disease (FMD)

**No**	**Authors**	**Year**	**Country**	**Study design**	**Study duration**	**Animal spp.**	**Breeds**	**Age group**
1	Bertrama et al	2018	Cameroon	Case control study	12-month	bovine	local Breed	Mixed
2	Soliman et al	2021	Egypt	Cohort study	4 weeks.	Ovine	local breed	Calves
3	Deghaid et al	2002	Egypt	Randomised experimental study	21 days	Bovine	Local breed	Calves
4	Al-Hosary A.A. et al"	2019	Egypt	Randomised controlled trial	1 year	Bovine	Local breed	Calves
5	Bazid et al	2023	Egypt	Randomised controlled trial	120 days	Bovine	local breed	Mixed
6	Shafik et al	2019	Egypt	randomized controlled study	35 days	Bovine	local breed	calves
7	Bagoury et al.	2013	Egypt	Cohort study	10 months	Calves	local Breed	Calves
8	Samy et al.	2021	Egypt	Randomised controlled trial	35 days	Bovine	local breeds	Calves
9	Eweis et al	2022	Egypt	Randomised controlled trial	40 days	Bovine	local breed	Calves
10	El-Deebb et al.	2017	Egypt	Randomised controlled trial	45 days	Bovine	Egyptian Baladi cattle	Calves
11	El-Sayed et al.	2012	Egypt	Randomised experimental study	40 Weeks	Bovine	Local breed	Calves
12	Mohamed et al.	2013	Kenya	Randomized controlled study	36 weeks	bovine and swine	local breed	calves
13	Sabenzia et al	2014	South Africa	Cohort study	April - may (3months)	cattle	local breed	Mixed
14	Scott et al	2017	South Africa	Cohort study	162 days	cattle	Nguni	Calves
15	Peta	2021	South Africa	Cohort study	18 months	Bovine and porcine	Nguni	Calves
16	CLOETE et al	2008	South Africa	Randomised controlled trial	1 year	Bovine	unknown	Adult
17	HUNTER	1996	Cameroon	Randomised controlled trial	7 months	bovine	Bonsmara cattle	Mixed
