# Supplementary material for: Foot and mouth disease vaccine efficacy in Africa: a systematic review and meta-analysis
Source: Front Vet Sci. 2024 Jun 6;11:1360256. doi: 10.3389/fvets.2024.1360256 (PMC11187330; doi:10.3389/fvets.2024.1360256)
Supplement: Supplementary file 2 [file Table_2.pdf]

Supp Table 2. Shows the quantitative information collected from 17 articles that met the criteria for inclusion. It summarises the experiment data collected from the articles including the total number of animal species, total number of vaccinated and unvaccinated animals, types of vaccines used for immunisation, circulating and vaccine serotypes and immune assay techniques.

**Authors**	**No. of samples**	**Total vaccinated**	**Total unvaccinated**	**Vaccinated protected**	**Type of vaccine**	**Vaccine serotype**	**Immune assay**	**Serotype in field**
Bertrama et al	150	50	100	(103/120)	Inactivated	O, A, SAT2	SNT	O, A, SAT_2
Soliman et al	32	30	2	(30/30)	Inactivated	SAT2, O, and A	SNT	SAT_2, O and A
Deghaid et al	32	27	5	(27/27)	Inactivated	O	Ab ELISA	O, A and SAT_2
Al-Hosary A.A. et al"	180	75	105	(58/75)	Inactivated	O serotype	SNT	O and SAT_2
Bazid et al	21	15	6	(15/15)	Inactivated	A, O and SAT2	VNT	O and SAT_2
Shafik et al	7	5	2	(5/5)	Inactivated	SAT2, O, and A	SNT	A, O and SAT_2
Bagoury et al.	150	150	0	(150/150)	inactivated	O and A	SNT and ELISA	A, O and SAT2
Samy et al.	12	10	2	(12/12)	Inactivated	O, SAT2 and A (Trivalent vaccine)	VNT	A and O

Eweis et al	30	20	10	(20/20)	Inactivated	A, SAT2 and O	VNT	AT2/EGY/2/2012 and SAT2, topotype VII, Lib-12 lineage (SAT2 Libya)
El-Deebb et al.	27	25	2	(14/15)	Inactivated	O Pan-Asia/2012	VNT	A, SAT2 and O
El-Sayed et al.	24	15	9	(10/10)	Inactivated	O Pan-Asia/2012	SNT and ELISA	A, SAT2 and O
Mohamed et al.	66	60	6	(18/20)	inactivated	A, and O	SNT and ELSIA	A and O
Sabenzia et al	191	42	149	(12/42)	Inactivated	A and O	ELISA	A and 0
Scott et al	23	21	2	(21/21)	reverse gentically constructed thermostabe vs Inactivated (wildtype)	SAT 2	ELISAs	SAT2 SAT1, SAT3
Peta	85	75	10	(58/75)	Inactivated	SAT2 of different 3 strains	ELSIA	SAT2 SAT1, SAT3
CLOETE et al	8	6	2	(4/6)	Inactivated	SAT1A, SAT1B, SAT2A, SAT2B, SAT3	ELSIA	SAT2 SAT1, SAT3
HUNTER	4	4	0	(3/4)	Inactivated	SAT1, SAT2 and SAT3	ELSIA SNT	SAT2 SAT1, SAT3
